# On Lateral Curvature of the Spine

**Published:** 1843-10

**Authors:** James Syme

**Affiliations:** Professor of Clinical Surgery in the University, Edinburgh


*On Lateral Curvature of the Spine.*—By JAMES SYME, Esq., Professor of Clinical Surgery in the University, Edinburgh.—[The object of this paper of Mr. Syme's is to point out the kind of case in which an operation for spinal curvature may be useful, and to discourage the operations which are recommended for all other kinds of spinal distortion. We cannot always be sure whether the bones or muscles are the first cause of lateral curvature. The muscles may be so weak as to allow the spine to bend, and the bones may at the same time be so unhealthy that an undue pressure may cause a premature distortion. All the operations which have been recommended for these lateral curvatures are at least very doubtful in their efficacy, if not positively injurious, and there is only one kind of spinal distortion which it is proper to attempt to remedy by subcutaneous incisions, and that is spinal curvature depending upon wry-neck caused by contraction of the sterno-mastoid, which muscle is liable to contraction both spasmodic and permanent. Although spasmodic contraction does not generally affect the shape of the spine, the permanent contraction may do so very materially.]

Wry-neck, like club-foot, is sometimes congenital, but much more frequently, like squinting, and the *pes equinus*, occurs during childhood, in connexion with some inflammatory or feverish state of the system. It depends upon a contracted state of the sterno-mastoid muscle, which has usually the feeling and appearance of a tense cord stretching from the clavicle to the ear. The head is bent towards the side affected, the face being turned to the opposite direction. Until the introduction of a subcutaneous incision, the treatment of this complaint was very defective, since it consisted either in the use of mechanical support, which did little, if any good, or in cutting across the contracted muscle, together with its superjacent integuments, which was a painful and bloody operation, leaving a large sore, slow to heal, and apt to renew the evil during cicatrization. The subcutaneous process requires merely a puncture of the skin, is not attended with pain or bleeding, needs no dressing or after-treatment, and at once affords the relief desired. The following is the first case on record of its performance in Great Britain, which I extract from my tenth report of the Edinburgh Surgical Hospital.

"*Wry-Neck depending on Contraction of the Sterno-Mastoid remedied by Division of the Muscle.*—Matthew Cullen, aged six, Dunbar, admitted November 2, 1832. The head was much inclined to the left side, and could not be elevated, owing to the rigid contraction of the left sterno-mastoid, the sternal part of which felt like a tense cord. The complaint had existed upwards of twelve months, and had resisted blisters, with other similar means of remedy. In these circumstances, it was thought necessary to divide the contracted part of the muscle. This was effected by entering a sharp-pointed narrow knife a little to the inner side of its tracheal margin, about an inch above the clavicle and then pressing the blade against the tense fibres. A sudden snap, which shook the patient's frame, was immediately perceived, and all trace of the contraction disappeared. The knife was withdrawn, and the small puncture occasioned by it in passing through the skin afforded the only perceptible indication of what had been done. No pain or other bad consequences followed, and the cure might be regarded as at once complete."—*Ed. Med. and Surg. Jour., p.* 115.

In the case just related, the disease existed in its simplest form, and without any affection of the spine, which is seldom wanting. It proceeds from involuntary elevation of the shoulder on the side affected, to lessen the strain on the head. This necessarily gives the dorsal part of the spine a corresponding convexity; and the lumbar portion bending in an opposite direction, to preserve the balance of the trunk, there results a lateral curvature, in no respect different, so far as regards appearance, from its ordinary condition. I was asked, along with Sir Charles Bell, to see a young gentleman suffering from lateral curvature. Finding the right sterno-mastoid very much contracted, I proposed to divide it, and did so with the effect of redressing the spine immediately, so that before we left the house the youth was walking straight.

But if the distortion be permitted to continue, it sooner or later, as in the ordinary form of the disease, leads to alteration in the shape of the bones. The bodies of the vertebræ are compressed, and the sternum projects. In this case, of course, the operation cannot afford the same instant relief that follows its performance while the curvature depends merely upon muscular action. The head, however, is at once set free, to the patient's great comfort; and through the gradual improvement of time, the trunk, unless arrived at full maturity, may in a great measure regain its proper conformation. A few weeks ago, I saw, with Mr. Cruickshank, of Hill Place, a young lady from this neighborhood, on whom I performed the operation in January, 1841. The back and ribs were then so much distorted, that I hardly ventured to hope for much improvement, especially as the patient was nearly twenty years of age. It was therefore an agreeable surprise to find, instead of the low, thin, sallow, crooked, sickly-looking girl I had formerly seen, an erect, fresh-colored, happy, healthy-looking young woman.

There is still another condition of the complaint, of which I may mention an instance that came within my notice last summer, in the case of a boy who was brought from the country on account of lateral curvature. Observing that his head inclined to one side, I examined the sterno-mastoid, and found it, not tense and rigid as I expected, but soft and yielding. I perceived, however, that when an attempt was made to raise the head, the muscle resisted and became tense, and therefore concluded that it was the seat of the evil. Under this impression I proceeded to divide it, and succeeded in doing so, though with more difficulty than usual, from the want of tension, for which it was necessary to compensate by stretching the neck. A good effect was immediately perceptible, and the following day the patient's back was comparatively straight, which it has since, I am informed, become completely.

In concluding these remarks, it may be well to warn against mistaking for wry-neck depending upon muscular contraction, the distorted position of the head which proceeds from caries between the occiput and atlas. The latter disease, like the former, usually occurs in young persons, presents to a careless observer similar symptoms, and if confounded with it, leads to treatment not only useless, but extremely injurious. A young gentleman had for twelve months used friction and exercise under the instruction of a distinguished member of the profession, who supposed that he labored under wry-neck from contraction of the sterno-mastoid. No benefit having been experienced, it was thought that an operation might be serviceable, and with this view I was asked to see the patient. He presented all the characters and well marked symptoms of *spondylarthrocace*, or caries at the occipito-vertebral articulation, in a stage so advanced, that there was nothing left for me but to explain the nature of the case and predict the fatal termination, which soon afterwards happened.—*Braithwaite's Retrospect of Med. and Surg.*, from *Lond. and Edin. Monthly Jour. Med. Sci.*, April 1843, p. 271.

